# Antiferromagnetic Ising model in a triangular vortex lattice of quantum fluids of light

**DOI:** 10.1126/sciadv.adj1589

**Published:** 2024-08-23

**Authors:** Sergey Alyatkin, Carles Milián, Yaroslav V. Kartashov, Kirill A. Sitnik, Ivan Gnusov, Julian D. Töpfer, Helgi Sigurðsson, Pavlos G. Lagoudakis

**Affiliations:** ^1^ Hybrid Photonics Laboratory, Skolkovo Institute of Science and Technology, Moscow, Territory of innovation center “Skolkovo,” Bolshoy Boulevard 30, bld. 1, Moscow 121205, Russia.; ^2^ Institut Universitari de Matemàtica Pura i Aplicada, Universitat Politècnica de València, 46022 València, Spain.; ^3^ Institute of Spectroscopy of Russian Academy of Sciences, Fizicheskaya Str., 5, Troitsk, Moscow 108840, Russia.; ^4^ Institute of Experimental Physics, Faculty of Physics, University of Warsaw, ul. Pasteura 5, PL-02-093 Warsaw, Poland.; ^5^ Science Institute, University of Iceland, Dunhagi 3, IS-107, Reykjavik, Iceland.

## Abstract

Vortices are topologically distinctive objects appearing as phase twists in coherent fields of optical beams and Bose-Einstein condensates. Structured networks and artificial lattices of coupled vortices could offer a powerful platform to study and simulate interaction mechanisms between constituents of condensed matter systems, such as antiferromagnetic interactions, by replacement of spin angular momentum with orbital angular momentum. Here, we realize such a platform using a macroscopic quantum fluid of light based on exciton-polariton condensates. We imprint all-optical hexagonal lattice that results into a triangular vortex lattice, with each cell having a vortex of charge *l* = ±1. We reveal that pairs of coupled condensates spontaneously arrange their orbital angular momentum antiparallel, implying a form of artificial orbital “antiferromagnetism.” We discover that correlation exists between the emergent vortex patterns in triangular condensate lattices and the low-energy solutions of the corresponding antiferromagnetic Ising system. Our study offers a path toward spontaneously ordered vortex arrays with nearly arbitrary configurations and controlled couplings.

## Teaser

Large-scale triangular lattice of coupled polariton quantum vortices approaches low-energy solutions of the Ising model.

## INTRODUCTION

Arrays of quantized vortices, appearing as phase-singular twists in wavefunctions, exemplify fascinating self-organizing phenomena, originally studied in macroscopic interacting Bose gases such as rotating superfluids and Bose-Einstein condensates displaying nucleation and ordering of single-charge vortices (*1*, *2*). More recently, optical vortex arrays in lasers have also gained increased interest (*3*–*6*) due to the promising applications of optical vortices (*7*) and phase-singular optics (*8*, *9*) in particle manipulation, high-resolution imaging, and quantum communication protocols (*10*, *11*). While optical systems offer superior spatial programmability over said vortex arrays (*6*), they do not have the large interaction strengths inherent to quantum fluids, which play a crucial role in phase transitions and pattern formation (*12*). For this reason, numerous studies have been focused on hybrid systems, which combine the best properties of light and matter to explore vortices (*13*). Here, we investigate such a light-matter platform in the strong-coupling regime based on cavity exciton-polariton (hereafter polariton) condensates (*14*), offering a compromise between optical programmability (*15*) and intrinsic evolution of vortex arrays in driven-dissipative quantum fluids (*16*–*20*).

Polariton condensates (*14*) are bosonic quantum fluids, characterized by large interaction strengths and light effective mass, that can be all-optically driven into artificial lattices using structured pump patterns (*21*–*23*). A notable feature of polariton condensates is their nonequilibrium superfluid character (*24*–*26*) and ability to form vortices that are pinned by sample disorder (*27*) or through optical trapping (*19*, *28*, *29*) or spun-up (*30*). To date, polariton vortex arrays have been observed in the interference pattern of multiple condensate modes (*17*, *20*) or in specially patterned cavities (*18*). However, large-scale programmable vortex lattices with tunable coupling strengths, where each vortex site can be individually addressed, remain unexplored in polariton fluids.

**Fig. 1.**
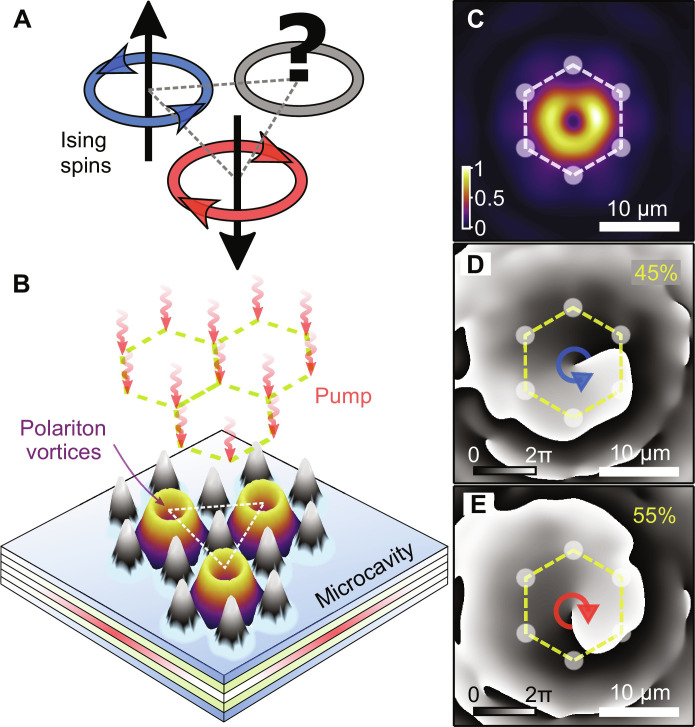
Driven trapped polariton condensates with OAM *l* = ±1 resembling classical spins. (**A**) Example of frustrated triangle for the Ising spins with AFM coupling. (**B**) Schematic of three polariton condensates in vortex states (yellow-violet surfaces), localized in the potential minima of the pump-induced energy landscape (black-gray surface). (**C**) Normalized experimentally measured time-averaged real-space polariton condensate photoluminescence for a single cell. (**D** and **E**) Examples of corresponding phase maps, revealing vortex and antivortex states, respectively. Dashed lines with circles schematically denote the excitation pattern. Numbers, in yellow, indicate statistical occurrence of states over 100 single-shot realizations.

As a particular example, we explore here the concept of driven-dissipative geometric frustration by designing a triangular lattice of “antiferromagnetically” (AFM) coupled polariton vortices. Here, AFM refers to polariton orbital angular momentum (OAM) instead of spin angular momentum. Conventionally, geometric frustration can be described as an inability of a system to minimize its real energy through reduction of pairwise interactions between constituent elements (*31*). A prominent example is Ising spins arranged into an AFM triangular graph (*32*), as shown schematically by the black arrows in Fig. 1A. Only two pairwise interactions can be minimized at any time, leaving the third at a higher energy. So far, artificial spin ice systems are popular candidates to explore frustration at large scale (*33*), but recently, lattices in bosonic systems have steered toward this direction using ultracold atoms (*34*), coupled lasers (*35*), and exciton-polariton condensates (*36*–*39*). However, instead of minimizing their real energy, polariton condensates minimize their losses (i.e., maximize their imaginary energy component) when they ballistically couple over mutually pumped region (*37*–*39*). From this perspective, one can define frustration for polaritons as their inability to minimize losses through non-Hermitian interactions between lattice nodes (*40*).

Here, we report on the evidence of frustration in a driven-dissipative triangular lattice of polariton condensates. To realize this, we optically imprint a lattice of trapped polariton condensates that populate a vortex state at each lattice node, as shown schematically in Fig. 1B. Instead of spin angular momentum, we work with condensates carrying OAM, with topological charges *l* = ±1, defined by a superposition of the trap first excited dipole modes ∣*P_x_*〉 ± *i*∣*P_y_*〉. We show that we can tune the coupling between synchronized vortex pairs to favor either parallel or antiparallel OAM. The latter constitutes an effective AFM coupling mechanism, which results in frustration when triangular geometry of condensates is introduced, due to incommensurate symmetries between the 𝒞_6_ (sixfold rotational symmetry of the triangular lattice) and SO(4) (rotation of the two-component OAM condensate) operators. Scaling up to a large 22-vortex system, we observe spontaneous formation of nonperiodic vortex configurations above threshold, changing from realization to realization, implying existence of multiple available modes. We provide experimental evidence that observed vortex patterns correlate notably with the ground-state solutions of the corresponding Ising system. The large nonlinearities inherent to exciton-polariton systems are crucial for the stability of the vortices, facilitating the readout of their OAM configurations even over long excitation pulses (≈10^7^ longer than the polariton lifetime).

## RESULTS

We use a planar 2λ GaAs-based microcavity with embedded InGaAs quantum wells (*41*) held at ≈4 K in a closed-cycle helium cryostat. Using a reflective phase-only spatial light modulator, we transform an incident nonresonant single-mode continuous-wave laser beam into an ordered honeycomb array (the dual of the triangular lattice) of small Gaussian beams focused onto the microcavity plane (see schematic in Fig. 1B) (*15*). The nonresonant circularly polarized pump is tuned at the first Bragg minimum of the microcavity reflectivity stop band to avoid heating of the sample (1.5578 eV). As we show in the Supplementary Materials, at the pump power used in experiments, trapped polariton condensate inherits the polarization state from the pump even under nonresonant excitation (*42*). This allows us to excite a single energy state corresponding to polaritons with a predominant pseudo-spin and make therefore a “clean” system without energy splitting (between σ^+^ and σ^−^ components).

The short distance between the pump spots (*d* = 6.6 μm) is chosen so as to obtain trapped polariton condensates in the center of each cell (i.e., in the region between six pumping spots) (*22*). Physically, polaritons become trapped in this region due to their strong repulsive interactions with the pump-injected background exciton reservoir (*43*–*45*). In note S1, we show that for larger honeycomb cells (*d* = 8.8 μm), the polaritons mostly condense on top of the pump spots instead of becoming trapped, underlining the importance of tuning the pump parameters.

### Vorticity onset in a single trap

To construct spatially extended triangular structure with coupled vortex states, we first implement and analyze their formation in a single, a pair, and a triangle of cells. In particular, the single-cell experiment allows us to confirm that the formation of vorticity is truly a spontaneous symmetry-breaking event with a random sign of vortex charge from realization to realization. Figure 1C shows time-integrated real-space polariton photoluminescence (PL) with a donut shape intensity profile for a single cell pumped at *P* = 1.2*P*_thr_.

The formation of a macroscopic wavefunction Ψ implies irrotational flow everywhere except at the vortex core, where the phase singularity is located and particle density vanishes. Phase winding around the core is quantized in units of 2π*l*, where *l* ∈ ℤ. To verify this, we apply a homodyne interferometric technique (*46*) to extract the phase of the condensate wavefunction under single-shot excitation conditions (i.e., the sample is excited by a single pulse of 50-μs duration). Our measurements of the single-shot phase maps across different realizations, where vortices form, reveal that 45% of the shots resulted in formation of a vortex (*l* = +1; Fig. 1D) and 55% in an antivortex (*l* = −1; Fig. 1E). Because of finite negligible pump anisotropy and sample disorder, we also find that 18 realizations out of 100 result in distributions with step-like π phase jumps, indicating the formation of a dipolar state, rather than of single vortex state, as it occurs in other 82 realizations, as described in note S2. The absence of a predominant sign of the observed vortex charges means that imprinted excitation pattern does not break chiral symmetry and that vorticity emerges spontaneously during the polariton condensation process.

In Materials and Methods, we show, using a simple variational generalized Gross-Pitaevskii model, that *l* = ±1 vortices in an ideal cylindrically symmetric optical trap (approximating the actual trap induced by six pump spots) are the only stable condensate solutions, in qualitative agreement with our observations. We emphasize that our nonresonant excitation pattern (shown in fig. S3) does not carry any OAM, nor does it imprint any phase onto the condensate, in contrast to resonant excitation schemes (*47*).

### Two coupled vortices

**Fig. 2.**
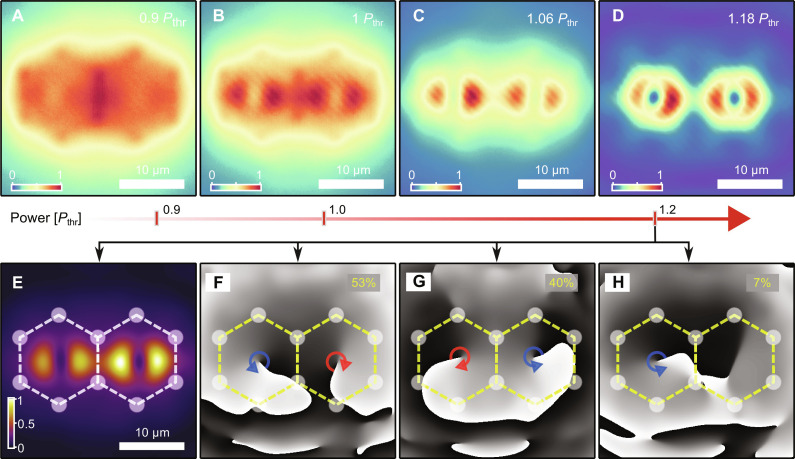
Vorticity onset and formation of vortex-antivortex pair in 2-cell structure. (**A** to **D**) Normalized experimentally measured time-averaged real-space polariton PL as a function of pump power. (A) Below and (B) at threshold, the polariton PL is mostly coming from the ridge of the pump-induced traps with no clear vortex formation. (C) At higher power, the condensate collapses inside the traps, forming first dipole-dipole state and then (D) a vortex-antivortex state. (**E**) Measured real-space polariton PL at *P* = 1.2*P*_thr_ and (**F** to **H**) corresponding examples of phase maps. Dashed lines with circles schematically denote the excitation pattern. Numbers, in yellow, in (F) to (H) indicate statistical occurrence of states over 100 single-shot realizations.

Next, we focus on two neighboring cells, shown in Fig. 2, which convincingly demonstrate signatures of coupling and synchronization. Figure 2 (A to D) shows measured time-averaged real-space polariton PL as a function of pump power. Clearly below (Fig. 2A) and at condensation threshold (Fig. 2B), the polariton PL does not reveal univocal signatures of vortex formation. However, at slightly higher power (*P* = 1.06*P*_thr_), the condensate collapses inside the traps forming dipoles (Fig. 2C), oriented head to tails (with parallel nodal lines) in a σ-bonding fashion as predicted recently (*48*). Then, above some vortex-threshold power, the condensates in both cells reveal a donut-shaped intensity distributions (Fig. 2D), indicating the onset of vorticity.

To verify that the coupling in this configuration is effectively AFM, we extract the phase maps of the vortex pairs at *P* = 1.2*P*_thr_ (see PL in Fig. 2E). Figure 2 (F to H) shows three typical examples of corresponding phase maps observed in a hundred independent single shots. Remarkably, we obtained that 93% of the condensate realizations formed with opposite OAM between the cells. The remaining 7% correspond to vortex-dipole (or antivortex-dipole) states. Therefore, our data analysis conclusively proves that the vortex coupling between cells strongly favors AFM order. Physically, this can be understood as an optimal constructive interference condition between the traps, which maximizes the gain of the condensate pair (*48*).

The advantage of our optical technique is that by changing some parameters of the pump profile, we can force the system to occupy vortex-vortex state (i.e., OAM “ferromagnetic” coupling) instead of vortex-antivortex states. To implement this, we optically imprinted weak potential barriers in the middle of the 2-cell structure in the same spirit as in demonstrated earlier coupling control approach (*46*). As a result, we managed to demonstrate reversible switching of the system from vortex-antivortex to vortex-vortex state as described in note S4.

### Three vortices in a triangle

**Fig. 3.**
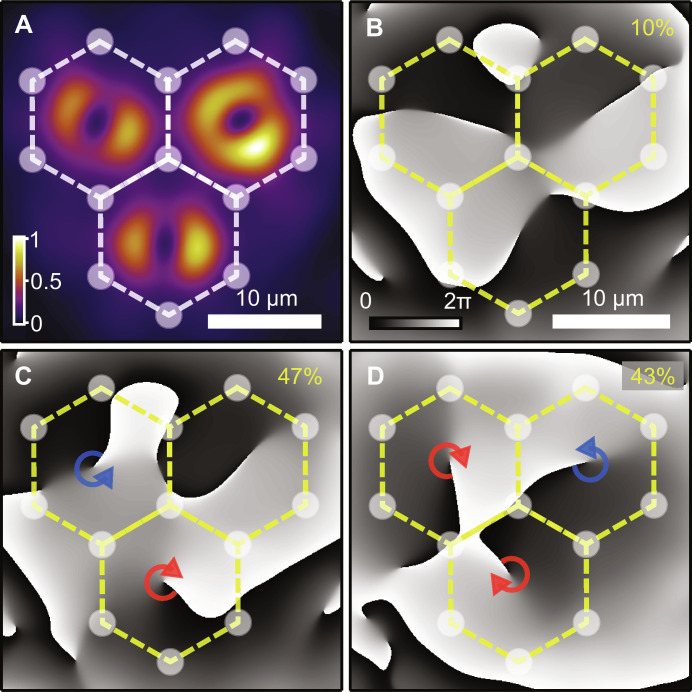
Vortex states in 3-cell structure. (**A**) Normalized experimentally measured time-averaged real-space polariton PL. (**B** to **D**) Corresponding extracted phase maps with numbers, in yellow, indicating statistical occurrence of states over 100 single-shot realizations. Dashed lines with circles schematically denote the excitation pattern.

Figure 3A shows the cavity PL after we optically construct an equilateral triangle of trapped condensates. The system’s preference for the AFM ordering as well as signatures of the expected topological vortex charge frustration is observed through homodyne interferometric measurements. Details on the interferometric technique are given in note S3 with an example of interference pattern given in fig. S5. In Fig. 3 (B to D), we provide few examples of extracted phase maps corresponding to different configurations, although the excitation conditions (geometry and pump power) are kept the same. For example, in Fig. 3B, one can see π phase jump lines implying formation of dipole states inside the cells in this particular realization. Dipole states can appear because of the sensitivity of polaritons to inherent cavity disorder or slight pump inhomogeneities, which pin the dipoles along a specific direction (*44*). We note that when a condensate is in a perfect superposition of vortex and antivortex modes, then the resulting dipole state has zero net OAM and therefore cannot be assigned to discrete variable of +1 or −1 following the Ising model. However, our further analysis indicates that when there is finite OAM in individual condensate sites, projecting it on classical binary variables shows pattern formation reminiscent of a frustrated triangular Ising system.

Nonetheless, for a major part of individual system’s realizations (47% out of 100 realizations), we find vortex-antivortex (antivortex-vortex) pair accompanied by a dipole state in the third cell, as shown in Fig. 3C. In 43% of condensate realizations, we reveal vortex-antivortex-vortex (or antivortex-vortex-antivortex) states in all cells, as depicted in Fig. 3D. We stress that we never observed vortices with the same OAM at the same time in all three cells. We also stress that Fig. 3 (C and D) shows examples over multiple possible arrangements of these type of patterns. These results, in the same vein as those of two cell structure, clearly indicate the tendency for development of the AFM configuration in each pair of neighboring cells. The fact that multiple realizations display a cell with an indeterminate vortex (see top right cell in Fig. 3C) implies dynamic indecisiveness reminiscent of frustration.

### Mean field modeling

**Fig. 4.**
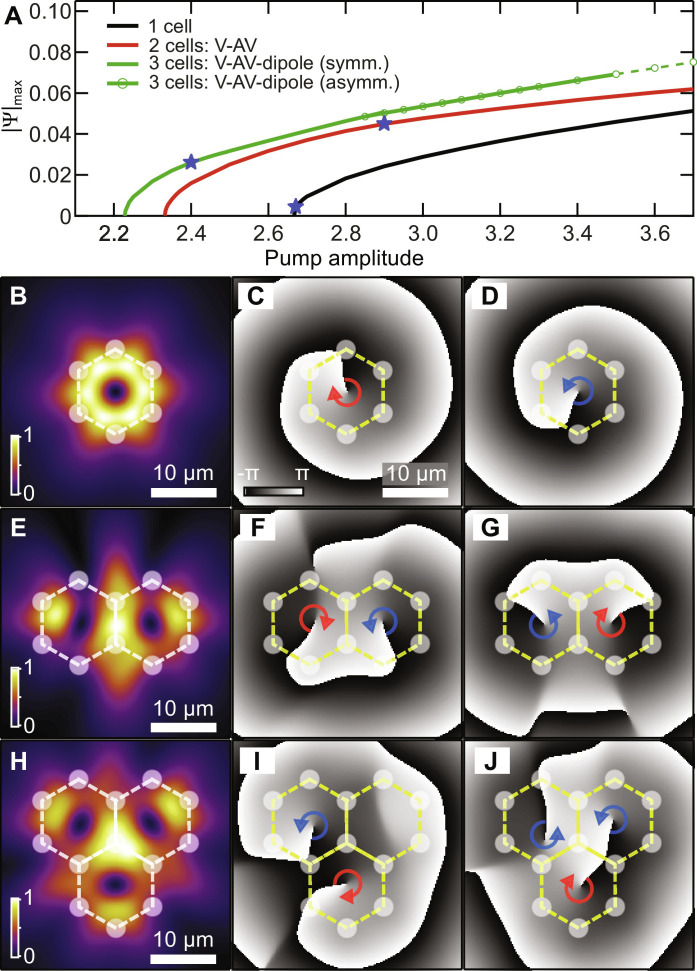
Vorticity dependence on pump power. (**A**) Calculated dependence of the stationary amplitude of the polariton wavefunction emerging in one, two, and three cells with varying pump power. The threshold (start of solid curves) lowers when more pump spots are introduced. The blue stars denote the onset of vorticity with increasing power. Below these stars, the polaritons dominantly localize on top of the pump spots with no apparent vorticity. For the 3-cell triangle (green curve), an additional asymmetric frustrated state appears (green circles) at higher powers. (**B** to **J**) Example solutions from 2DGPE simulation of the condensate density and phase in building blocks of the lattice showing agreement with experiment.

Our experimental observations can be reproduced through numerical mean-field simulations, based on the generalized two-dimensional (2D) Gross-Pitaevskii equation (2DGPE) describing a macroscopic polariton wavefunction, Ψ(**r**, *t*), coupled to the rate equation for the exciton reservoir, *n_X_*(**r**, *t*) (see Materials and Methods). First, we analyze the vorticity onset in the cells of the lattice. To obtain families of possible solutions (modes) corresponding to one-, two-, and three-cell pump configurations, we apply the Newton method and perform linear stability analysis. Families of stationary states Ψ(**r**, *t*) = *w*(**r**)*e*^−*i*ε*t*^ with ∂*_t_n_X_* = 0 (here, ε is the energy detuning) that emerge as stable attractors around threshold correspond to the colored curves in Fig. 4A. In this graph, we show the dependence of the scaled peak amplitude of the wavefunction ${\left(g_{c}m_{\text{eff}}r_{0}^{2}/\hbar^{2}\right)}^{1/2}|\Psi |$ on pump power *P*_0_. Here, *r*_0_ = 1 μm is the characteristic spatial scale, *m*_eff_ is the polariton mass, and *g_c_* is the polariton-polariton interaction strength. One can see that the condensation threshold power (starting point of the curves) decreases with increasing number of pumped cells as expected (*49*).

On each solid curve, the onset of vorticity with pump power (i.e., finite net clockwise or anticlockwise rotation) is indicated with a blue star. For a single honeycomb pump cell (black curve), a stable vortex emerges almost at condensation threshold, in good agreement with experiments. For the two-cell configuration (red curve), we find that the stable branch emerging from threshold corresponds to vortex-antivortex pairs forming at a critical power given by the blue star, while below this power vorticity is absent and field distribution exhibits dipole structure. This critical pump power represents a vorticity threshold, observed experimentally in Fig. 2 (C and D). When we add a third pump cell, the first stationary family emanating from threshold is associated with a symmetric vortex-antivortex-dipole configuration appearing at the blue star on the green curve in Fig. 4A, in agreement with observations in Fig. 3 (A and C). For lower powers (below blue star), the condensate resembles a triple dipole state, whereas for high powers it gradually transforms into an asymmetric vortex-antivortex-dipole solution (solid green curve with dots). Even further increase of pump power results in unstable behavior (denoted with green dashed curve in Fig. 4A) and the emergence of different stationary states *w*(*r*), such as the antivortex-vortex-antivortex shown in Fig. 3D. Corresponding solutions around the blue stars on these three branches are shown in Fig. 4 (B to J) obtained from direct numerical integration of the 2DGPE using white noise initial conditions and damped boundary conditions.

In Materials and Methods, we additionally develop a simplified model describing the coupling between vortices by treating them as localized orbitals in each trap (i.e., tight binding approach). This reduces the problem of modeling a 2 + 1 nonlinear partial differential equation into a set of 2*N* ordinary differential equations (Eq. 6). Here, *N* is the number of traps and the factor “2” appears because of clockwise and anticlockwise currents. This effective “spinor” model allows us to analytically prove the stability of vortices in isolated traps through construction of a Lyapunov function. It also correctly predicts increased power of the vortex threshold when two traps are coupled together, in agreement with experiment and 2DGPE simulations. One advantage of the model (Eq. 10) is that it has analogies with conventional spinor polaritons in patterned cavities with effective spin-orbit coupling (subject to future work).

### Triangular lattice of AFM-coupled vortices: Evidence of low-energy Ising order

**Fig. 5.**
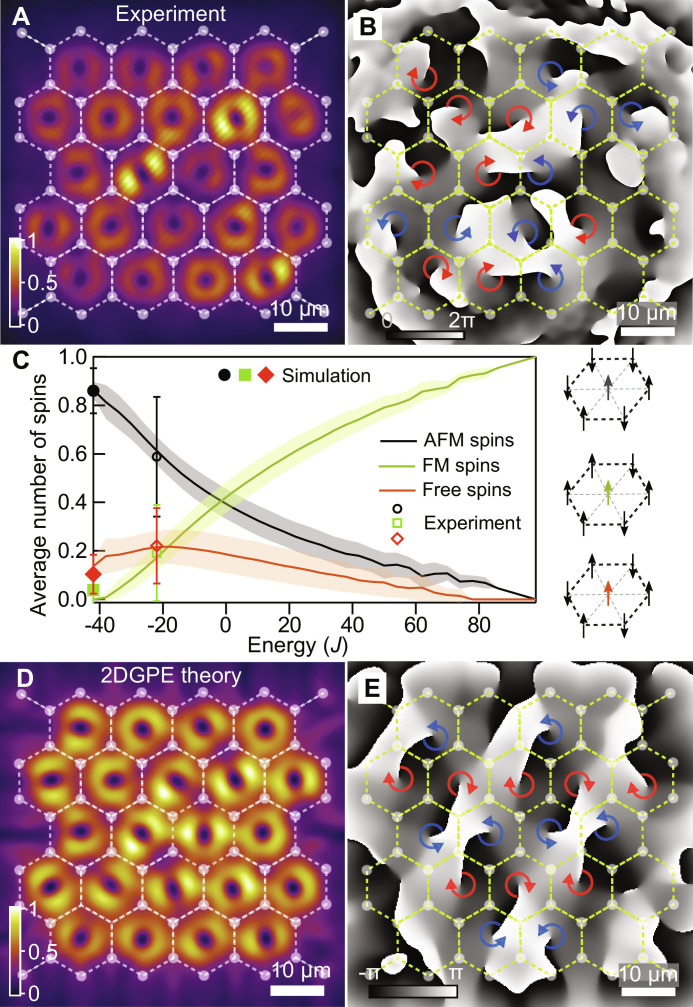
Vorticity dependence on pump power. (**A**) Experimental time-integrated real-space polariton PL intensity (above condensation threshold) and (**B**) measured single-shot realization of the condensate phase illustrating the formation of a large-scale vortex lattice. (**C**) Calculated normalized mean number of AFM, FM, and free spins in the 22-spin AFM triangular Ising lattice as a function of energy. The shaded areas correspond to one standard deviation (SD). The three types of spins are schematically depicted on the right. The average vortex populations obtained from experiment are plotted with the empty markers, with error bars denoting SD. The obtained average populations from a discretized Gross-Pitaevskii simulations are shown with filled black, green, and red markers and, remarkably, correspond to the minimum energy *E* = −42*J* (see note S6 for details on model and calculations). Overlaid honeycomb lattice in (A), (B), (D), and (E) schematically shown with dashed lines and semi-transparent circles indicates the pump positions. Blue and red arrows show schematically the phase winding direction in each cell. (**D**) Time-averaged realization of the condensate density above threshold reveals donut-shaped PL. (**E**) Instantaneous condensate phase obtained from mean field modeling confirms vortex formation.

Finally, we study the vorticity patterns appearing in much larger spatially extended structures, consisting of 22 cells in a triangular geometry. Figure 5A shows polariton condensation into a macroscopic coherent state containing multiple vortices across the lattice for a pump power above condensation threshold unlike previous observations in square (*15*, *37*, *50*), triangular (*15*), and Lieb (*22*) optical lattices. Similar to our observations in smaller structures, each cell has a donut-shaped PL intensity profile.

Figure 5B shows extracted from experiment a single-shot phase map with multiple single-charge (*l* = ±1) phase singularities across the lattice, schematically marked by blue and red arrows. We underline that the excitation pulse width (50 μs) is much longer than the cavity lifetime (≈5.5 ps), indicating the topological robustness of the vortices. We observe that across multiple realizations (provided in note S5) the pattern of vortices changes as one might expect due to the spontaneous symmetry breaking upon condensation.

The apparently random configurations of vortices from realization to realization would suggest that the system is completely stochastic, which would be the case if the traps are very weakly coupled. However, as we know from observations on the two-cell (Fig. 2) and three-cell (Fig. 3) experiments, the traps are AFM-coupled, which should manifest in some form of AFM order in the lattice OAM with a highly degenerate “ground state” in analogy to Ising systems (*32*).

To address this question, we analyzed the spatial OAM order of the vortices appearing in 25 single-shot realizations of the 22-cell structure, and checked for correlations with the low-energy configurations of the Ising Hamiltonian (see note S8 for derivation of its role in picking optimal vortex arrangements)

$$E=-J\sum_{\langle n,m\rangle }‍\sigma_{n}\sigma_{m}$$ (1)

where *J* < 0 is the AFM coupling energy, the variables σ*_n_* ∈ {±1} represent Ising spins, and the sum is taken over nearest neighbors in the triangular lattice. The up/down OAM of each lattice node is then projected onto its corresponding Ising spin and classified into one of three groups (*32*): (i) spin has more co-aligned neighbors (“FM” spins, *E* is raised), (ii) spin has more counter-aligned neighbors (“AFM” spins, *E* is lowered), and (iii) spin has equal co- and counter-aligned neighbors (“free” spins, *E* is unchanged). Here, we refer the reader to small insets in Fig. 5C, on the right. The ground state of Eq. 1 can be organized in many different ways using conditionally ordered AFM spins and free spins.

All 2^22^ possible spin configurations and associated energies from Eq. 1 can be easily found by brute force. Binning each spin of each configuration into one of the three possible categories described above, we can plot the mean and standard deviation (SD) of the normalized number of spins in each category as a function of Ising energy *E* (see curves and shaded areas in Fig. 5C). As expected, the highest energy state has all spins parallel (only FM spins) with zero SD, whereas the ground state has only AFM and free spins with finite SD (implying degeneracy). We then apply the same classification to the vortices in our experimental data (see fig. S8) and extract corresponding statistical occurrence of FM, AFM, and free vortices normalized against the number of detected vortices and plot on top of Fig. 5C. The three data points (as black, green, and red empty markers) are placed where their error is minimum from the solid curves, which puts them at *E* = −22*J*. Remarkably, our data points are within the lower ≈6% of Ising spin configurations from the ground state. From this, we conclude that our vortex lattice does not display stochastic arrangement of vortices, which would give *E* = 0, but instead tends toward AFM order. Corresponding simulations from a variational discrete Gross-Pitaevskii simulation (see details in note S6) are able to locate the Ising ground state as shown by filled markers in Fig. 5C. 

Figure 5 (D and E) shows a representative example of the simulated condensate density and phase distributions emerging from random initial conditions, evidently illustrating the formation of a vortex lattice. Excitation from random noise typically yields dynamical, breathing, structures that nevertheless are characterized by persistence of vortices, once they form in the cells. In agreement with the experiment, the arrangement of vortices changes from simulation to simulation (see more examples in fig. S9), illustrating the highly multimodal nature of the lattice, yet maintaining a dominant AFM order across the 22-cell structure.

## DISCUSSION

We note that self-arranging vortices are well known in rotating superfluids (*51*), atomic Bose-Einstein condensates (*2*), and nonlinear optical systems (*3*, *4*, *6*, *7*). Here, we provide evidence that our all-optical lattice supports large-scale polaritonic modes (or super-modes), which arise due to the intercell interactions in our nonlinear system, rather than to a collection of uncoupled individual single-site states. As such, system states are prone to develop strong instabilities that could demolish their robustness and, hence, their observation. For this reason, the observation of a large number of coupled vortices constitutes in itself a remarkable finding, which could not be anticipated a priori. With 22 cells (Fig. 5, A and B), a large number of stationary states coexist for pump powers just above the condensation threshold and the system exhibits a large degree of multistability. As a consequence, the different experimental realizations with 22 cells reveal the formation of a wide diversity of states where individual vortex locations appear stochastically from one realization to the next.

Future investigations could verify whether it is possible to excite a macroscopic polariton system in a superposition of eigenstates carrying the vorticity and provide a clear answer to a fundamental question: Is stochasticity of observed vortex states in such a system a solid proof of its multistability? In general, interferometry cannot unambiguously prove that the system is not in a quantum superposition of states within the same energy manifold. Therefore, to verify the hypothesis of a superposition of eigenstates, one would need to implement quantum vortex tomography, which requires experimental single-shot realization of a vortex sorter in free space and temporal correlation measurements with high resolution. The systematic study of extended lattices of coupled vortices, although challenging, offers a promising avenue of research with an outlook to quantum simulators that requires thorough theoretical consideration.

In conclusion, we demonstrate evidence of an all-optically imprinted lattice of interacting vortices in nonresonantly driven-dissipative quantum fluids of light. Whereas in a single cell, we observe an intrinsically stochastic behavior of an optically trapped polariton condensate, in the presence of the second neighboring cell we reveal synchronization into a vortex-antivortex state, resembling the OAM analog of AFM coupling. We also analytically predicted and experimentally demonstrated optical control of the vortex interactions from AFM to FM. Therefore, by tuning the interactions between ordered trapped polariton condensates, one can expect observation of various artificial magnetic phases between the nodes, emulating complex magnetic systems, and the study of other exotic coherent states in expanded structures. Our findings address the challenge of realizing the node-to-node coupling in spatially confined quantum fluids of light possessing OAM.

## MATERIALS AND METHODS

### Generalized Gross-Pitaevskii model

The condensate dynamics is assumed to be described by the 2D generalized Gross-Pitaevskii equation (2DGPE) for the polariton wavefunction Ψ(**r**, *t*) coupled to a rate equation describing the exciton reservoir feeding the condensate *n_X_*(**r**, *t*) (*52*)

$$\mathrm{i\hbar}\frac{\partial \Psi }{\partial t}=\left\{-\frac{\hbar^{2}\nabla^{2}}{2m_{\text{eff}}}-i\frac{\hbar }{2}\left(\gamma_{c}-Rn_{X}\right)+g_{c}{\left|\Psi \right|}^{2}+g_{r}\left[n_{X}+\eta P\left(\text{r}\right)\right]\right\}\Psi$$ (2)

$$\frac{\partial n_{X}}{\partial t}=-\left(\gamma_{X}+R{\left|\Psi \right|}^{2}\right)n_{X}+P\left(\text{r}\right)$$ (3)

Our parameters are based on the sample properties and past experiments (*41*). Here, *m*_eff_ ≈ 5.63 × 10^−5^*m_e_* is the effective mass of the lower-branch polaritons, where *m_e_* is the free electron rest mass, *g_c_* = 2.4 μeVμm^2^ is the polariton-polariton interaction strength, and *g_r_* = 2*g_c_* is the polariton-reservoir interaction strength typical for GaAs-based systems; *R* = 0.021 μm^2^ps^−1^ is fitted rate of stimulated scattering of polaritons from active reservoir; γ*_c_* ≈ 0.182 ps^−1^ and γ*_X_* ≈ 0.05 ps^−1^ are the decay rates for polariton condensate and reservoir excitons, respectively; ηγ*_X_* ≈ 0.49 quantifies an additional blueshift coming from a background of dark reservoir excitons. The function *P*(**r**) = (*P*_0_γ*_X_*γ*_c_*/*R*)∑*_n_* *Q*(**r** − **r***_n_*) describes spatial pump profile consisting of identical Gaussian spots *Q*(**r**) = *e*^−*r*^2^/*d*^2^^ of width *d* (corresponding to 2.5-μm-wide pump spots used in the experiment) placed in the points with coordinates **r***_n_* defining particular pump configuration (one or several honeycomb cells or large-scale honeycomb lattice with period *D*). Dimensionless pump amplitude *P*_0_ is defined here in units of γ*_X_*γ*_c_*/*R* corresponding to condensation threshold for spatially uniform pump.

The system of Eqs. 2 and 3 were solved using a variant of split-step fast Fourier transform method combined with fourth-order Runge-Kutta method to account for interaction between the condensate and the reservoir. To uncover all existing stationary states in the system and possible types of evolution, including essentially dynamical regimes, when excitation of stationary states does not occur, the modeling was initiated using different realizations of small-scale noise for initial Ψ, *n_X_* distributions. To identify most of all possible stationary solutions of the system or dynamical evolution regimes, multiple (up to several hundreds) input noise realizations were implemented for each pump power and pump configuration. To account for considerable ballistic expansion of polaritons from the pumped regions leading to nonzero polariton density even far from them, we used in modeling sufficiently large spatial domain 300 × 300 μm^2^ greatly exceeding the pumped area. Stationary states of Eqs. 2 and 3 corresponding to ∂*_t_n_X_* = 0 were found in the form Ψ(**r**, *t*) = *w*(**r**)*e*^−*i*ε*t*^, where *w*(**r**) is a complex function describing stationary polariton distribution in the cavity plane and ε is the energy detuning, using Newton iteration method. Because of the dissipative nature of our system, the energy detuning ε of stationary states, some of which appear as stable attractors, is also determined by the pump amplitude *P*_0_. Stability of such stationary states, whose families are presented in Fig. 4 for different pump configurations, were tested by modeling their evolution in Eqs. 2 and 3 and by performing linear stability analysis on them.

### Single-condensate vorticity onset

Here, we will write a variational form of the generalized Gross-Pitaevskii model describing only the dynamics of the first excited degenerate pair of *l* = ±1 OAM modes in the condensate. We will then proceed to prove, for an ideal cylindrically symmetric trap, that the only (and equally) stable condensate solutions correspond to these OAM modes.

We will assume for simplicity that the continuous wave–driven excitonic reservoir follows the condensate adiabatically so that ∂*_t_n_X_* ≃ 0, which implies $n_{X}=\frac{P\left(r\right)}{\gamma_{X}+R{\left|\Psi \right|}^{2}}\approx \frac{P\left(r\right)}{\gamma_{X}}\left(1-\frac{R{\left|\Psi \right|}^{2}}{\gamma_{X}}\right)$ . In the last step, we have Taylor expanded the reservoir solution around small *R*|Ψ|^2^/γ*_X_*, which is valid at pump powers not too far from threshold. This allows us to write a simpler 2DGPE

$$\begin{array}{cc} \mathrm{i\hbar}\frac{\partial \Psi }{\partial t}= & \left[-\frac{\hbar^{2}\nabla^{2}}{2m_{\text{eff}}}+g_{c}{\left|\Psi \right|}^{2}-\frac{\mathrm{i\hbar}\gamma_{c}}{2}\right.\\ & \;\;\left.+g_{r}\eta P\left(r\right)+\left(g_{r}+i\frac{\mathrm{\hbar R}}{2}\right)\frac{P\left(r\right)}{\gamma_{X}}\left(1-\frac{R{\left|\Psi \right|}^{2}}{\gamma_{X}}\right)\right]\Psi \end{array}$$ (4)

We are interested in a condensate that occupies the degenerate pair of clockwise and anticlockwise OAM states. Assuming an ideal cylindrically symmetric trap *P*(**r**) = *P*(*r*), we can write our truncated basis as

$$\Psi \left(r\right)=\xi \left(r\right)\left(\psi_{+}e^{i\theta }+\psi_{-}e^{-i\theta }\right)$$ (5)

Here, ξ(*r*) is the radial steady state (∂*_t_*|Ψ|^2^ = 0) part of the condensate in a single optical trap by solving the 2DGPE. Plugging in this truncated solution into Eq. 4 and integrating over the space, exploiting the orthogonality of the states, we arrive at two coupled equations of motion describing the vortex phase and occupation

$$i\frac{d\psi_{\pm }}{\mathrm{dt}}=\left[i\tilde{p}+\left({\tilde{g}}_{c}-i\tilde{R}\right)\left({\left|\psi_{\pm }\right|}^{2}+2{\left|\psi_{\mp }\right|}^{2}\right)\right]\psi_{\pm }$$ (6)

up to an overall energy shift. Note the factor 2 in the counter-rotating nonlinearity (cross-Kerr term), which is orders of magnitude larger than the singlet interaction strength in spinor polariton condensates. The new coefficients are given by $\tilde{p}=\frac{P_{0}\gamma_{c}}{2}\int ‍\xi {\left(r\right)}^{2}Q\left(r\right)\;dr-\frac{\gamma_{c}}{2}$, ${\tilde{g}}_{c}=\frac{g_{c}}{\hbar }\int ‍\xi {\left(r\right)}^{4}\;dr-\frac{P_{0}g_{r}\gamma_{c}}{\hbar \gamma_{X}}\int ‍\xi {\left(r\right)}^{4}Q\left(r\right)\;dr$, and $\tilde{R}=\frac{P_{0}R\gamma_{c}}{2\gamma_{X}}\int ‍\xi {\left(r\right)}^{4}Q\left(r\right)\;dr$ . Equation 6 can be written in terms of the amplitude and phase of each mode $\psi_{\pm }=\sqrt{N_{\pm }}e^{i\phi_{\pm }}$

$$\frac{dN_{\pm }}{\mathrm{dt}}=2\left(\tilde{p}-\tilde{R}N_{\pm }-2\tilde{R}N_{\mp }\right)N_{\pm }$$ (7a)

$$\frac{d\phi_{\pm }}{\mathrm{dt}}={\tilde{g}}_{c}\left(N_{\pm }+2N_{\mp }\right)$$ (7b)

We see that the change in the phase of the modes is trivially determined by the dynamics of their amplitudes. We therefore only need to focus on solutions of Eq. 7a, which has three equilibrium points: *N*_±_ = 0 and $N_{\mp }=\tilde{p}/\tilde{R}$ ; and $N_{+}=N_{-}=\tilde{p}/3\tilde{R}$ . It is easy to show that the only (and equally) stable equilibrium points are the former through the eigenvalues λ of the Jacobian of Eq. 7a. This can also be visualized by plotting the system Lyapunov potential with extrema corresponding to these solutions

$$ℒ=-2\tilde{p}\left(N_{+}+N_{-}\right)-\tilde{R}\left(N_{+}^{2}+N_{-}^{2}\right)-4\tilde{R}N_{+}N_{-}$$ (8)

**Fig. 6.**
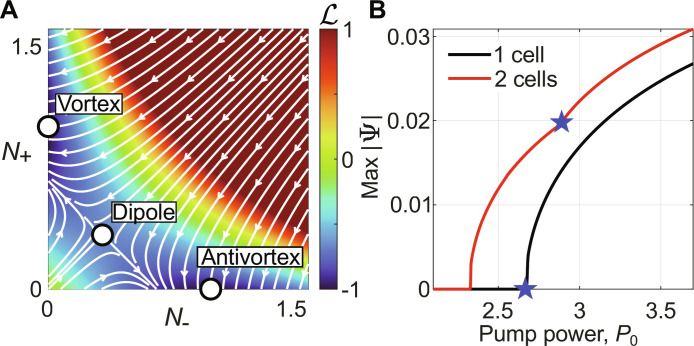
Vorticity onset in the single and pair of trapped condensate cells. (**A**) The color scale depicts the Lyapunov potential (Eq. 8) of the system, with two minima (white dots) corresponding to vortex and antivortex solutions in which all phase space trajectories converge toward, underlining the deterministic nature of the system. Here, we set $\tilde{p}=\tilde{R}=1$ without loss of generality. (**B**) Reproduced results from Fig. 4A using the variational Gross-Pitaevskii model for a single (Eq. 6) and a pair of coupled condensates (Eq. 10). The blue stars denote the onset of vorticity for increasing power.

The Lyapunov potential satisfies the condition *dℒ*/*dt* ≤ 0, which means that all points in phase space flow toward the two minima indicated by the white dots in Fig. 6A corresponding to the two opposite vortex solutions. The unstable saddle point extremum, corresponding to a dipole solution, is also clearly visible. Our simple model evidences that the only stable condensate solutions in a single cell are vortices, which is in agreement with experiment.

These vortex solutions bifurcate from the uncondensed normal phase *N*_±_ = 0 at condensation threshold of the single trap written

$$P_{0,\text{thr}}^{1\text{cell}}=P_{0,\text{vort}}^{1\text{cell}}={\left[\int ‍\xi {\left(r\right)}^{2}Q\left(r\right)\;dr\right]}^{-1}$$ (9)

The above integral quantifies the amount of overlap between the condensate and the pumped region. If this overlap increases, then the threshold is lowered as expected.

### Vorticity onset for two coupled condensates

The equations describing two coupled trapped condensates read

$$\begin{array}{c} i\frac{d\psi_{n,\pm }}{\mathrm{dt}}=\left[i\tilde{p}+\left({\tilde{g}}_{c}-i\tilde{R}\right)\left({\left|\psi_{n,\pm }\right|}^{2}+2{\left|\psi_{n,\mp }\right|}^{2}\right)\right]\\ \psi_{n,\pm }+J_{a}\psi_{3-n,\pm }+J_{b}\psi_{3-n,\mp } \end{array}$$ (10)

where *n* = 1,2 denote the left and right condensate. Here, *J*_*a*,*b*_ ∈ ℂ are the non-Hermitian tunneling rates between co-rotating and counter-rotating vortices (i.e., OAM conserving and nonconserving coupling strengths, respectively)

$$J_{a,b}=\left(\frac{g_{r}\left(1+\gamma_{X}\eta \right)}{\hbar }+i\frac{R}{2}\right)\frac{P_{0}\gamma_{c}}{R}I_{a,b}$$

where

$$I_{a,b}=\int ‍\xi {\left(r\right)}^{*}e^{\mp i\theta }Q_{\text{tot}}\left(r\right)\xi \left(r\prime \right)e^{i\theta \prime }\;dr,\in \mathbb{R}$$ (11)

Here, *Q*_tot_(**r**) describes the two pumped traps and the separation between them can be written $r\prime -r=d\hat{x}$ . The coordinates of the two pumps are related through $r\prime =|r\prime |=\sqrt{r^{2}+d^{2}-2\mathrm{rd}\text{cos}\left(\theta \right)}$ and sin(θ**′**) = *r* sin (θ)/*r***′**.

Physically, Re(*J*_*a*,*b*_) and Im(*J*_*a*,*b*_) correspond to a Josephson (particle conserving) and dissipative (particle nonconserving) coupling, respectively, between the condensates. Calculating Eq. 11 numerically using the single-cell wavefunction found from 2DGPE simulations, we find that *I*_*a*,*b*_ < 0, which means that Im(*J*_*a*,*b*_) < 0 and therefore the dissipative coupling favors anti-phase synchronization between neighboring condensates (see note S7). This is in agreement with observations in experiment (Fig. 2, E to H) and 2DGPE simulations (Fig. 4, E to G). The negative value of the integral intuitively makes sense because anti-phase displaced vortices constructively interfere between the two cells. Thus, anti-phase condensates overlap more strongly with the pumped region between the traps and are populated more efficiently.

The lowest threshold solution of the coupled system (Eq. 10) is then written $\psi ={\left(\psi_{1,+},\psi_{1,-},\psi_{2,+},\psi_{2,-}\right)}^{T}=\sqrt{N}{\left(1,1,-1,-1\right)}^{T}e^{-i\omega t}$ with occupation $N=\left[\tilde{p}+|\text{Im}\left(J_{a}+J_{b}\right)|\right]/3\tilde{R}$ and frequency ω = 3*N* + ∣Re (*J_a_* + *J_b_*)∣. This is an antiphase dipole-dipole orientated head to tails (∞) ↔ (∞) with a condensation threshold of ${\tilde{p}}_{\text{thr}}=-|\text{Im}\left(J_{a}+J_{b}\right)|$ , in agreement with previous predictions (*48*). Such anisotropic low-threshold solution intuitively makes sense since polaritons flow stronger out of the trap parallel to the condensate dipole axis, thus creating optimal coupling (overlap) conditions between two spatially interacting condensates (e.g., similar to σ-bonding between atoms).

At higher pump powers, these dipoles in each condensate loose stability and bifurcate each into either *l* = ±1 vortices. When ∣*I_b_*∣ > ∣*I_a_*∣, the system favors AFM ordering, like reported in Fig. 2 (E to H). If ∣*I_b_*∣ < ∣*I_a_*∣, the converse is true and FM ordering is dominant (which we show experimental evidence of in the Supplementary Materials). This vortex transition point $P_{0,\text{vort}}^{2\text{cells}}$ can be analytically derived for the AFM case ∣*I_b_*∣ > ∣*I_a_*∣ (the FM case follows similar treatment) by locating the onset of linear instability for the dipole-dipole solution

$$\begin{array}{c} P_{0,\text{vort}}^{2\text{cells}}=\\ \frac{1+\frac{4g_{c}^{2}\left(1+\gamma_{X}\eta \right)}{R^{2}}\left[2-\frac{\gamma_{X}}{\gamma_{c}}\frac{\int ‍\xi {\left(r\right)}^{4}\;dr}{\int ‍\xi {\left(r\right)}^{4}Q\left(r\right)\;dr}\left(I_{a}+I_{b}\right)\right]}{I_{p}\left(1+\frac{8g_{c}^{2}\left(1+\gamma_{X}\eta \right)}{R^{2}}\right)+\left[2I_{a}+3I_{a}{\left(\frac{4g_{c}\left(1+\gamma_{X}\eta \right)}{R}\right)}^{2}-I_{b}-2\frac{4g_{c}^{2}\left(1+\gamma_{X}\eta \right)}{R^{2}}\left(I_{a}+I_{b}\right)\right]} \end{array}$$ (12)

Note that *I*_*a*,*b*_ < 0, which means that if the coupling anisotropy *I_b_*/*I_a_* is large, then the critical power of the AFM vortex formation reduces. This means that by tailoring the pump *P*_tot_(**r**), one can adjust *I*_*a*,*b*_ to shift the position of the transition point.

We obtain good results shown in Fig. 6B using *I*_*a*,*b*_ as fitting parameters and solving the variational Gross-Pitaevskii equation for a single cell (Eq. 6) and two cells (Eq. 10) as a function of increasing power. Our results are in excellent agreement with 2DGPE simulations previously shown in Fig. 4A, and thus explain the essential observations of our experiment. The blue stars indicate the onset of vorticity in the system. Going further, we have also extended Eq. 10 to the case of arbitrarily coupled cells and analyzed the onset of vorticity and the preference toward AFM order when ∣*I_b_*∣ > ∣*I_a_*∣ in the 3- and 22-cell configuration (see note S6).

## Supplementary Materials

### This PDF file includes:

Supplementary Notes S1 to S8 Figs. S1 to S11
