# Supplementary material for: Antiferromagnetic Ising model in a triangular vortex lattice of quantum fluids of light
Source: Sci Adv. 2024 Aug 23;10(34):eadj1589. doi: 10.1126/sciadv.adj1589 (PMC11343025; doi:10.1126/sciadv.adj1589)
Supplement: Supplementary file 1 — Supplementary Notes S1 to S8 Figs. S1 to S11 [file sciadv.adj1589_sm.pdf]

Science Advances
AAAS

Supplementary Materials for

Antiferromagnetic Ising model in a triangular vortex lattice of quantum fluids of light

Sergey Alyatkin *et al.*

Corresponding author: Sergey Alyatkin, s.alyatkin@skoltech.ru;
Pavlos G. Lagoudakis, pavlos.lagoudakis@gmail.com

Sci. Adv. **10**, eadj1589 (2024)
DOI: 10.1126/sciadv.adj1589

This PDF file includes:

Supplementary Notes S1 to S8
Figs. S1 to S11

SUPPLEMENTARY NOTE 1: LATTICE CONSTANT DEPENDENCE

In Fig. S1 we show the real-space polariton photoluminescence (PL) excited nonresonantly in fragment of honeycomb lattice. When the lattice constant is set to $D = 15.3$ µm, we observe formation of ballistically coupled polariton condensates, co-localised with Gaussian pumps as visible from Fig. S1A. For smaller lattice constant set to $D = 14.1$ µm we clearly observe repulsion of polaritons outside the pumped spots, which leads to formation of triangular-shaped (Fig. S1B) polariton condensates above threshold. Finally, when $D = 11.5$ µm, our time-integrated measurements reveal formation of large-scale vortex lattice, shown in Fig. 5A of the manuscript.

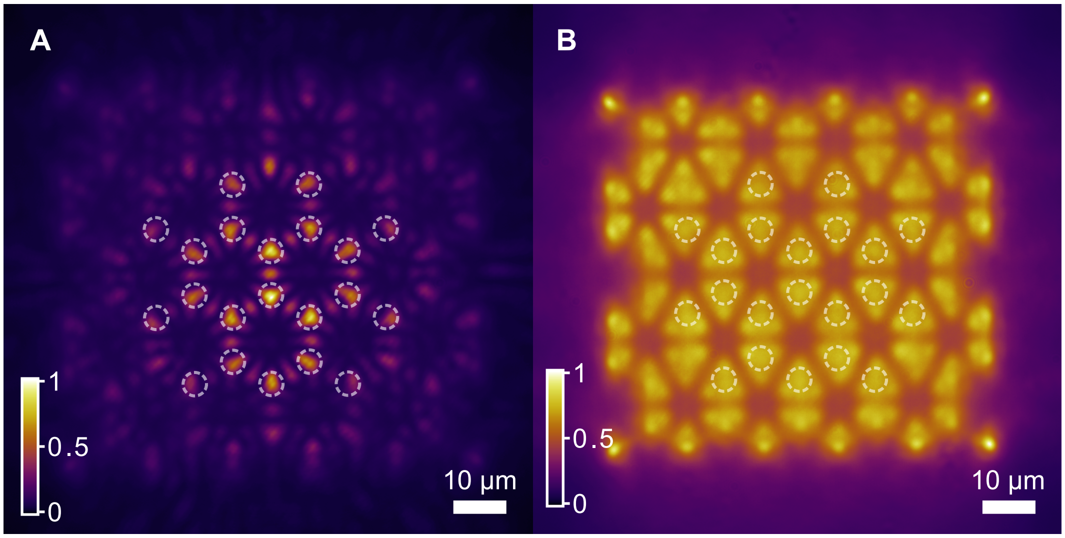

Figure S1. **Lattice constant dependence.** (**A**),(**B**) Experimentally measured time-averaged polariton photoluminescence intensity distribution for the lattice pumped above condensation threshold, and lattice constant set to $D = 15.3$ µm and $D = 14.1$ µm, respectively. In (**A**) the condensates form on top of the pumping spots and synchronize across the lattice, while for smaller lattice constant in (**B**) the polaritons repelled outside pumped areas form triangular-shaped condensates. White dashed circles schematically denote pumped spots, forming honeycomb lattice.

SUPPLEMENTARY NOTE2: PUMP PROFILE USED IN EXPERIMENTS WITH "BUILDING BLOCKS" OF THE LATTICE

In order to realize a truly spontaneous formation of the vortex states in one-, two- and three-cell structures, we use active feedback technique that allows for equalizing the integrated intensities of the pump spots with a typical achievable precision (standard deviation) below 2%. For this we real-time measure a corresponding pump profile, extract pumps intensities and iteratively recalculate a phase mask, applied to spatial light modulator (SLM), to reach desired uniformity of the pump pattern. When succeeded, the calculation stops and the SLM phase mask becomes static (i.e. no longer updated). At this stage all experiments for specific structure are conducted: time-integrated PL measurements and single-shot homodyne interferometry. This approach ensures that the excitation laser profile remains exactly the same throughout measurements. As an example in Fig. S2A we show obtained pump profile used in experiments with a single honeycomb cell. The statistical occurrence for vortex and antivortex states, shown in Fig. 1D and Fig. 1E, convincingly proves that pump profile is uniform to avoid pump-induced vorticity with preferable topological charge. However, out of 100 single-shot realizations we observed also 18 realizations of the dipole states (see Fig. S2B), which could appear due to sample disorder and/or finite imperfections of pump profile (though balanced in intensity). The numbers, in yellow, shown in Fig. 1D and Fig. 1E of the manuscript are renormalized such as to show statistical occurrence out of 82 realizations.

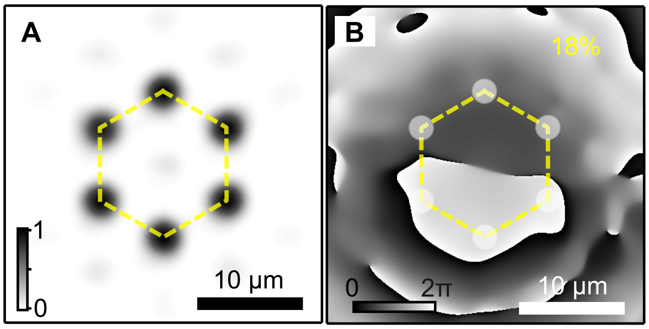

Figure S2. **Nonresonant excitation profile for a single cell and an example of a dipole state.** (**A**) Measured laser pattern with balanced intensities of the spots and (**B**) measured single-shot phase map, corresponding to a dipole state (observed in 18 realizations out of 100). Dashed lines and semi-transparent white circles schematically denote pump spots.

To confirm that selected number of one hundred shots is reasonable enough for statistics to provide a trustful distribution of cases occurrence, we analyzed 401 single-shot realizations for 2-cell system shown in Fig. 2 of manuscript and obtained very similar occurrence (90.8% vs 93% obtained for 100 shots) for realizations with opposite OAM. The rest 9.2% correspond to observation of vortex-dipole (or antivortex-dipole) states. All nonresonant pump profiles used in the experiments with one-, two- and three-cell structures are shown in Fig. S3. The characteristic size of the unit cells is kept the same ($D = 11.5$ μm).

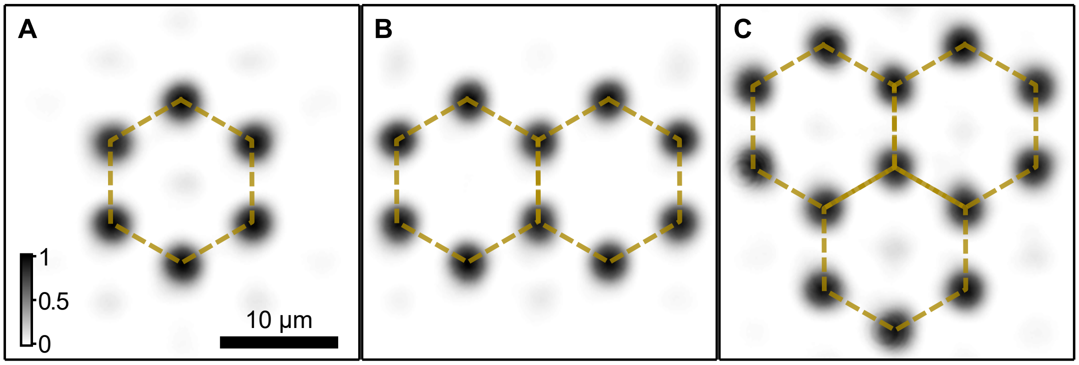

Figure S3. **Nonresonant excitation profile for "building blocks" of the lattice with constant set D = 11.5 μm.** Normalized experimentally measured laser profile used to excite vortices in one (**A**), two (**B**) and three (**C**) honeycomb cells, corresponding to experimental data shown in Fig. 1C,D,E, Fig. 2 and Fig. 3 of the manuscript. Dashed lines schematically denote the excitation pattern to guide the eye. Scale bar in (**A**) applies to all panels.

SUPPLEMENTARY NOTE 3: HOMODYNE INTERFEROMETRY TECHNIQUE

In this work we use nonresonant continuous wave (CW) excitation laser, spatially shaped according to desired excitation pattern (number of nodes, their diameter, separation distance and relative intensities). In order to reconstruct a real-space polariton PL intensity and the corresponding phase map of the condensate state from measured single-shot interference pattern (obtained with a homodyne interferometry), we apply the off-axis digital holography technique.

Below we briefly describe the principle of homodyne interferometry method. First, in addition to acousto-optically modulated CW laser, we use a weak CW seed resonant laser, synchronously modulated to the nonresonant pump laser. In all our experiments, described in the manuscript, we used circularly polarized laser for optical excitation. As we show in Fig. S4, at pump power used in the experiments, a condensate trapped inside a hexagonal cell inherits the polarization from the pump even under nonresonant excitation. This allows us to excite a single energy state corresponding to polaritons with a single predominant pseudo-spin and make therefore, a "clean" system without any energy splitting (between σ^+ and σ^- polaritons). The seed laser, tuned to the energy of the condensate, is focused (FWHM $\approx$ 2 µm) on the sample through the same microscope objective as the main excitation pattern, and essential only to locally fix the phase of the condensate with respect to the reference wave. Second, the collected polariton PL, is interfered with a flat reference wave in Mach-Zehnder interferometer. The reference wave originates from the same laser (with narrow linewidth of $\approx$ 100 kHz) as the seed. Third, the interference pattern (see Fig. S5A) is recorded with a sensitive CCD camera and analyzed using off-axis digital holography. The resultant real-space phase and intensity maps are shown in Fig. S5B,C.

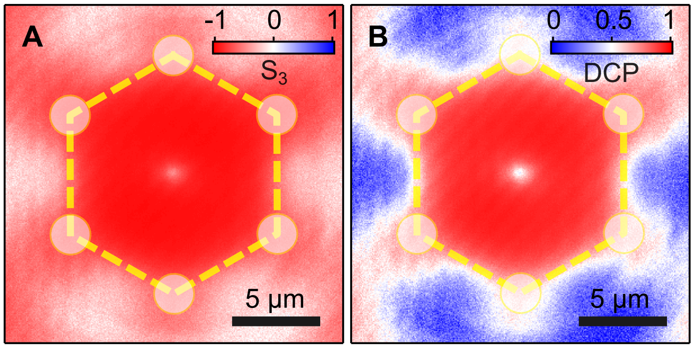

Figure S4. **Polarization-resolved polariton photoluminescence in a single cell.** (**A**) Experimentally measured map of the Stokes parameter S_3 for the trapped condensate excited with CW circularly polarized laser emission. Corresponding degree of circular polarization (DCP) map in **B** confirms that the condensate is mostly circularly polarized. Dashed lines with circles schematically denote the excitation pattern.

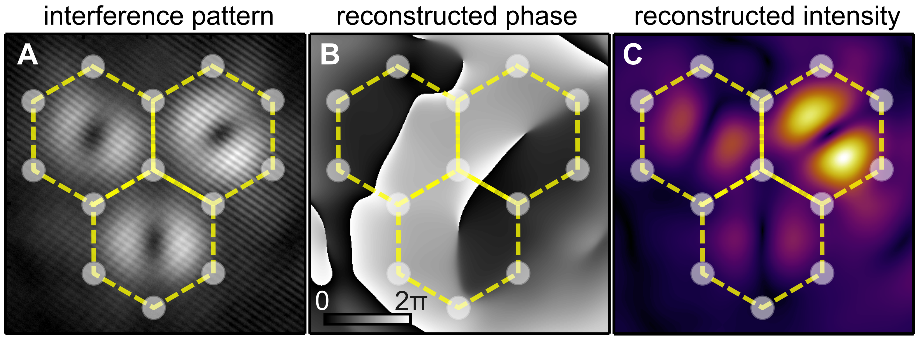

Figure S5. **Analysis of example single-shot interference pattern for 3-cell configuration.** (**A**) Recorded interference pattern, (**B**) corresponding reconstructed phase map and (**C**) reconstructed intensity map. Semi-transparent white circles denote pump positions to guide the eye.

Figure S6 shows different examples of experimentally measured real-space phase maps corresponding to predicted families of the states observed in 3-cell structure (shown in Fig. 3 of the manuscript). The numbers here correspond to statistical occurrence of the states over one hundred individual realizations. We note that only in 7% of cases (7

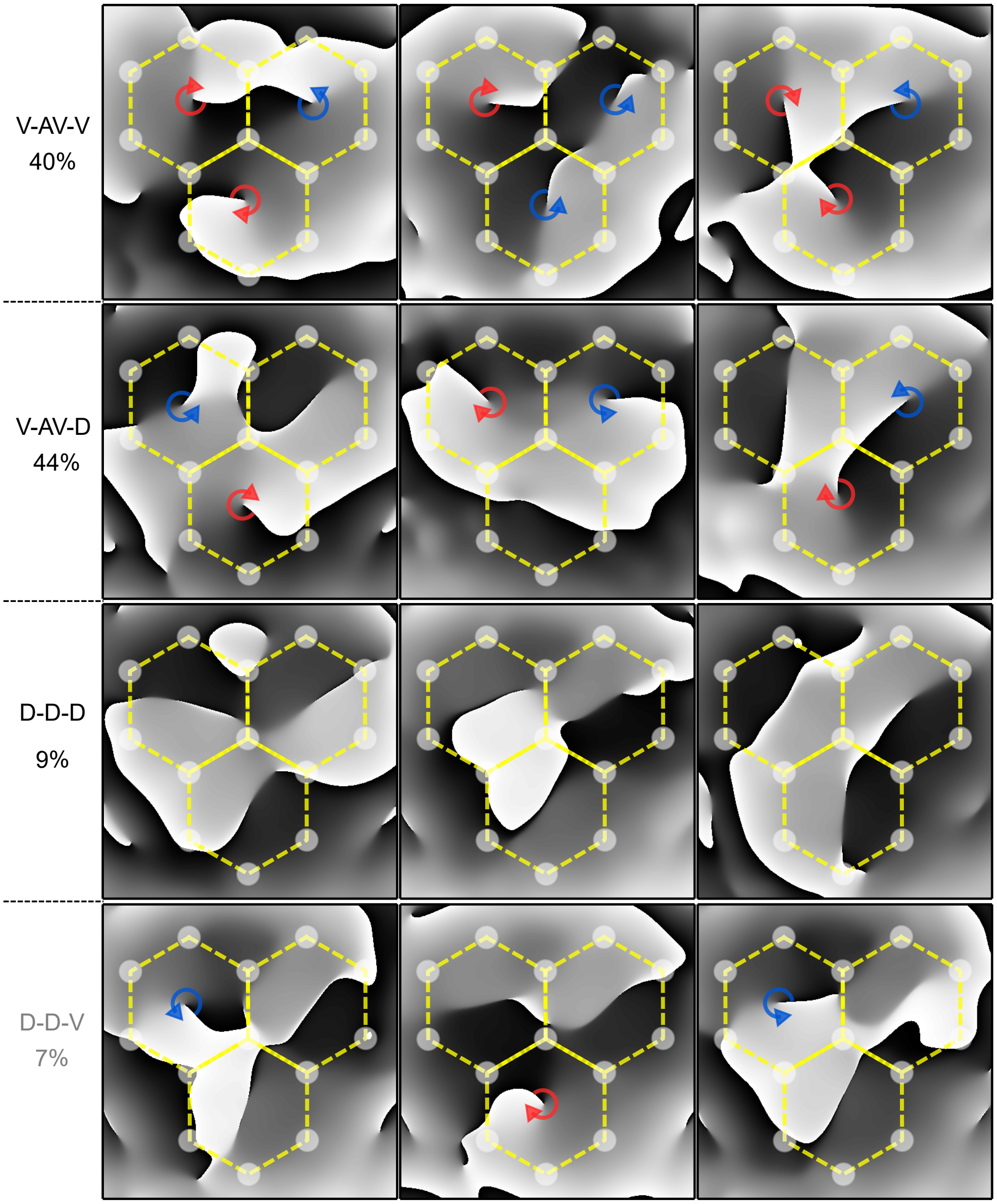

Figure S6. **Examples of the reconstructed phase maps corresponding to different families of the states observed in 3-cell structure.** The number in % shows corresponding statistical occurrence based on analysis of one-hundred single shot interference patterns. Semi-transparent white circles denote pump positions to guide the eye. V, AV and D stands for vortex, antivortex and dipole states, respectively.

realizations) we classified the state as dipole-dipole-vortex (bottom panels in Fig. S6). We believe this state can be explained by some tiny imperfections of the pump or sample disorder given the nonlinear nature of exciton-polariton system. The rest of the families are in a good agreement in our theoretical prediction. The numbers, in yellow, shown in Fig. 3B-D of the manuscript are renormalized such as to show statistical occurrence out of 93 single-shot realizations.

SUPPLEMENTARY NOTE 4: INTERACTION CONTROL IN COUPLED CELLS

Our experimental measurements of the single-shot realizations in 2-cell structure revealed that such system preferentially occupies vortex-antivortex or antivortex-vortex states. However, none of the realizations demonstrated the same orbital angular momentum (OAM). At the same time, our developed model predicts that by changing the coupling between the cells one can potentially tune the system to vortex-vortex state, implying FM order instead of dominant AFM arrangement. To realize this experimentally, we first reproduce already expected vortex-antivortex pair using pump profile shown in Fig. S7A. As a result, above condensation threshold power we observe formation donut-shaped PL inside the cells (Fig. S7B), corresponding to a vortex-antivortex pair as confirmed by the extracted phase map in Fig. S7C.

To change the coupling between the trapped synchronized states we additionally optically inject three nonresonant weak excitation beams, as shown in Fig. S7D (see also inset on the right with the line profiles of the condensate pumps and barriers pumps). We note, that for the barrier pattern we use cross-circularly polarized excitation with respect to the condensate pumps. This allows to minimize gain due to overlap of the condensate wave functions with the barrier potentials in the middle of the 2-cell structure. It turns out, that even when the brightest spot of the barrier pattern (Fig. S7D) is ≈17 times less intense ($I_0/17$) than average integrated intensity I_0 of the condensate pump (Fig. S7A), the initial system state definitely switches to vortex-vortex, as shown in Fig. S7E,F. By this we mean that in none of the analyzed 100 single-shot realizations we observed states with opposite OAM of polaritons in the traps, once the barrier pump overlaid with condensate pump. Such an all-optical approach with structured excitation to control the vortices interactions opens a new route towards engineering the macroscopic coherent states with desired spatial distribution of OAM.

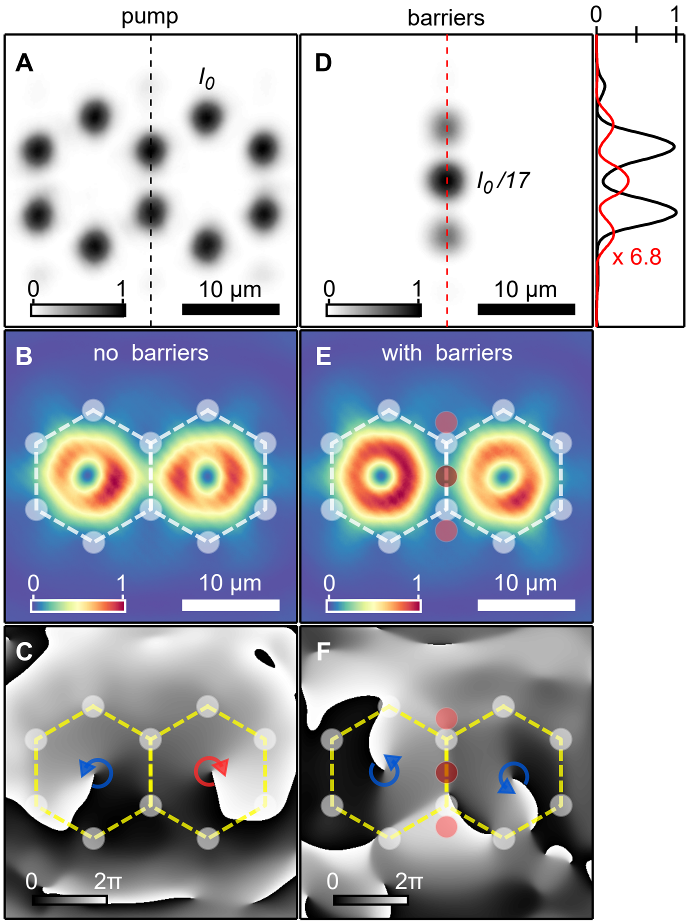

Figure S7. **Switching the vortex interactions by means of weak optically imprinted barriers in between the cells.** (**A**) Normalized nonresonant pump profile and (**B**) corresponding time-averaged polariton PL, demonstrating formation of (**C**) vortex-antivortex pair. When the barriers pump in (**D**) additionally injected, the system switches to vortex-vortex state as visible from PL intensity in (**E**), and single-shot pahse map in (**F**). White circles schematically denote the condensates pump positions, red circle denote barriers positions.

SUPPLEMENTARY NOTE 5: EXPERIMENTAL EVIDENCE OF “ORBITAL ANTIFERROMAGNETISM” ORDERING IN 22-CELL STRUCTURE

Figure S8 shows examples of extracted from experiment single-shot phase maps corresponding to the state of 22-cell structure, shown in Fig. 5A of the manuscript. With dashed lines and semi-transparent circles we schematically denote nonresonant pump pattern. Each shown phase map reveals numerous vortices (follow blue arrows) and antivortices (follow red arrows). The total number of phase dislocations associated with vortices and antivortices, localized inside the honeycomb cells, is written in the right top corner, in orange, as the second number. The first written number, in orange, shows the sum of classified AFM and free nodes/vortices (by analogy with spins) as described in the main manuscript. For visual perception, in each cell with vortex or antivortex we place a star with a colour, corresponding to spin category (see small insets at the bottom in Fig. S8). The analysis clearly indicates that dominant portion of cells with vortices demonstrate AFM coupling (i.e. major part on neighbours has opposite measured OAM of polaritons). Totally, we analyzed 25 single-shot realizations with the results plotted in Fig. 5C as empty markers. Remarkably, such approach allows to experimentally evidence the presence of dominant AFM order across different realizations with lacking any order at first glance.

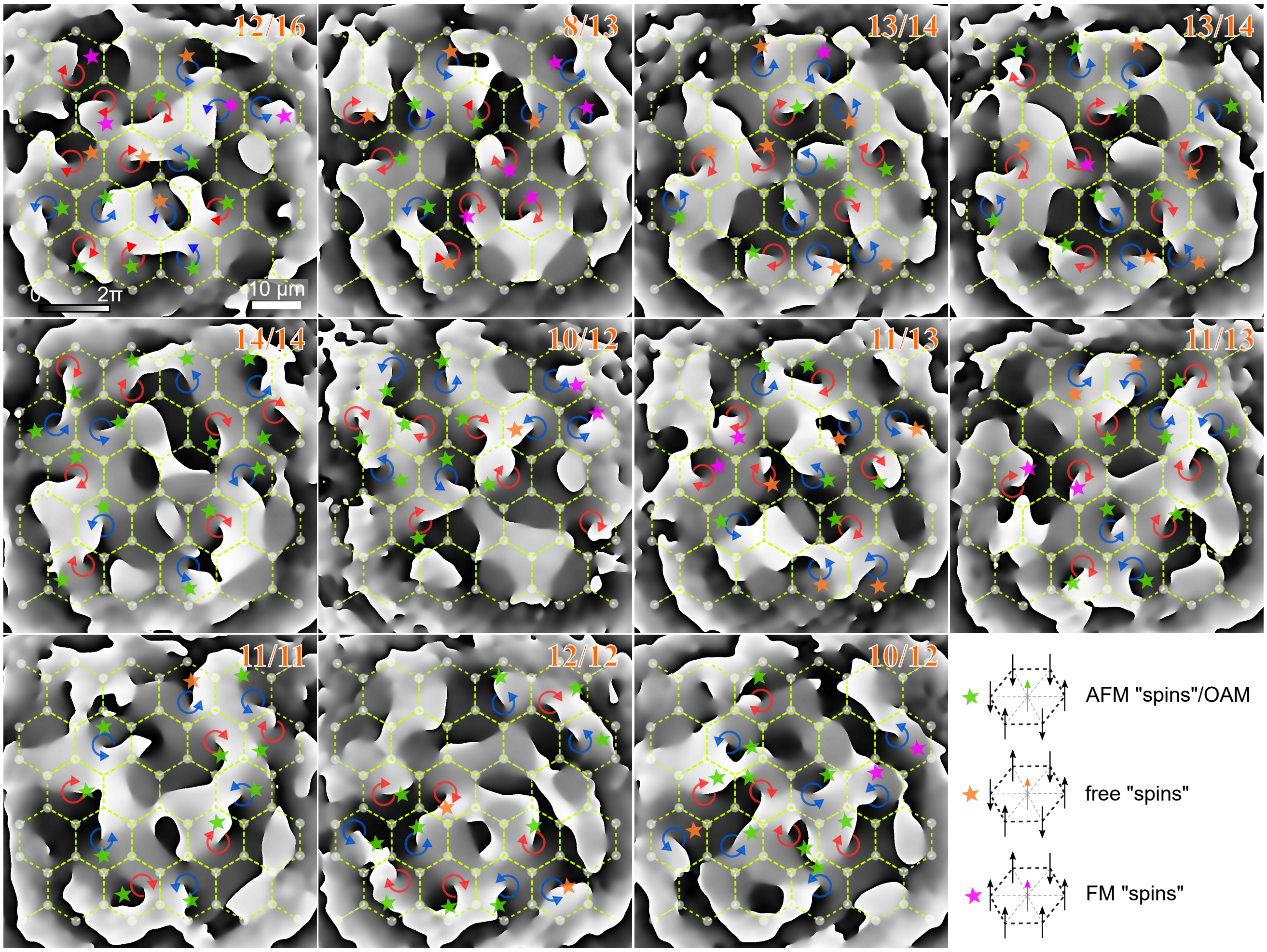

Figure S8. **Experimentally extracted single-shot phase maps of the polariton condensate in 22-cell structure with lattice constant set $D = 11.5$ μm.** Blue and red arrows schematically indicate vortices and antivortices. Each lattice cell with vortex/antivortex inside is classified according to the description given in the main manuscript and marked by a star. Green star corresponds to “spin”(vortex) which has AFM coupling with neighbours. Orange star corresponds to free “spin”, or total zero OAM of polaritons in neighboring cells. Magenta star corresponds to “spins” with dominantly co-aligned neighbours (FM spin). Dashed lines with semi-transparent white circles denote pump pattern to guide the eye.

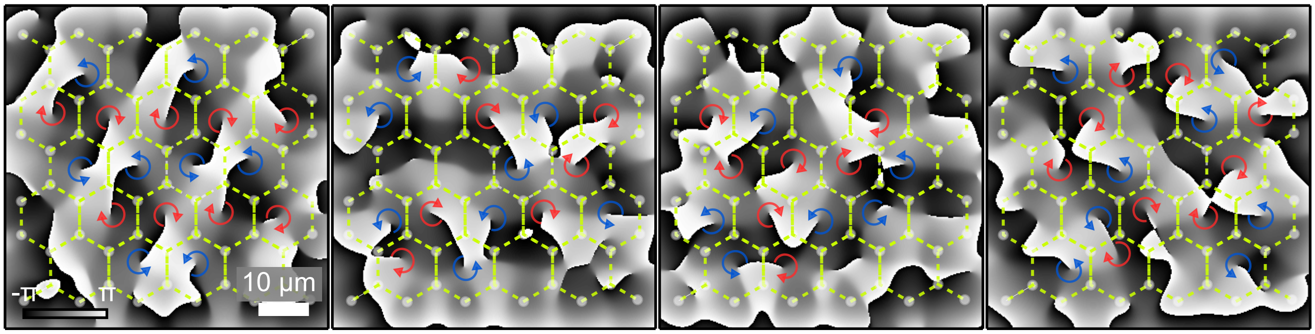

Figure S9. **Simulated single-shot realizations of the condensate phase map for the 22-cell structure.** Dashed lines with semi-transparent white circles schematically denote pump pattern.

Figure S9 shows simulated instantaneous phase maps, corresponding to Fig. 5D of the manuscript. Similar to experimental observations in Fig. S8 the vortices stochastically flip their topological charges from realization-to-realization, yet maintaining the dominant AFM order across 22-cell structure. In simple words, the polaritons OAM in the given cell strongly depends on the neighbouring cells.

SUPPLEMENTARY NOTE 6: VARIATIONAL GROSS-PITAEVSKII MODEL FOR COUPLED POLARITON VORTICES

Extending Eq. (10) in the main text to arbitrary geometries of coupled nearest-neighbour polariton vortices gives,

$$i\frac{d\psi_{n,\pm}}{dt} = \left[i\tilde{p} + (\tilde{\alpha} - i\tilde{R})(|\psi_{n,\pm}|^2 + 2|\psi_{n,\mp}|^2)\right]\psi_{n,\pm} + \sum_{\langle n,m\rangle}\left[J_a\psi_{m,\pm} + J_b\psi_{m,\mp}e^{\mp 2i\Theta_{n,m}}\right]. \tag{S1}$$

Here, $\psi_{n,\pm}$ is the phase and amplitude of the nth condensate component with OAM $l = \pm 1$, $J_{a,b} \in \mathbb{C}$ are the tunneling rates between co-rotating and counter-rotating vortices, $\tilde{\alpha}$ corresponds to the repulsive polariton-polariton interactions, $\tilde{R}$ represents a gain saturation mechanism in the adiabatic exciton-reservoir limit, and $\tilde{p}$ is the combined non-resonant optical pumping rate and cavity losses. The sum runs over nearest neighbours and $\Theta_{n,m}$ is the angle of the link between two condensates in separate traps [notice the double winding in the exponent of Eq. (S1)] [47]. In the special case of triangular geometry we have $\Theta_{n,m} \in \{0, 2\pi/3, 4\pi/3\}$.

A. 3-cell system: AFM order in a single triangle

Here we numerically investigate the presence of AFM order in a single triangle when the power parameter $\tilde{p}$ is scanned. It is convenient to characterise the behaviour of the three-cell system by defining a three-dimensional Bloch vector (or pseudospin) for each condensate similar to what is done in optics with light beams carrying OAM,

$$S^n_{x,y,z} = \begin{pmatrix}\psi^*_{n,+} & \psi^*_{n,-}\end{pmatrix}\hat{\sigma}_{x,y,z}\begin{pmatrix}\psi_{n,+}\\ \psi_{n,-}\end{pmatrix}, \tag{S2}$$

where $\hat{\sigma}_{x,y,z}$ are the three Pauli matrices. Physically, projection on the $S^n_{x,y}$ components means that the nth condensate has some dipolar structure. Projection on the S^n_z component means that the nth condensate has vorticity. Specifically, $S^n_z > 0$ corresponds to a counterclockwise vortex and $S^n_z < 0$ to a clockwise vortex.

We numerically solve Eq. (S1) for the triangle of traps, averaging over 1000 random initial conditions. We show in Fig. S10A the normalised time-average and cell-average projection of the condensates on the dipole states $\sqrt{\langle S_x^2\rangle + \langle S_y^2\rangle}$ and vortex states $\sqrt{\langle S_z^2\rangle}$ where,

$$\langle S^2_{x,y,z}\rangle = \frac{1}{3}\sum_{n=1}^{3}\frac{1}{T}\int_0^T \left(S^n_{x,y,z}\right)^2 dt, \tag{S3}$$

as a function of pump power $\tilde{p}$ scaled in units of $|\text{Re}(J_a)|$. Here we set $\text{Re}(J_{a,b}) < 0$ and $\text{Im}(J_{a,b}) < 0$ and $|J_b|/|J_a| = 1.5$ in agreement with the overlap integral calculation shown in Fig. S11. We choose $\text{Im}(J_{a,b})/\text{Re}(J_{a,b}) = 0.5$, and $\tilde{\alpha} = 0.1\tilde{R}$. We point out that our results do not depend strongly on the choice of parameters as long as $\text{Im}(J_a) < \text{Im}(J_b) < 0$.

From an optics perspective, these quantities are also similar to a light source's degree of linear polarization (DLP) and degree of circular polarization (DCP) except now for OAM. We also show in Fig. S10B the "amount" of AFM order using the following order parameter,

$$\langle M \rangle = \sum_{n<m} \frac{1}{T} \int_0^T S_z^n S_z^m \, dt. \tag{S4}$$

Namely, if the order parameter $\langle M \rangle < 0$ then the system is preferentially AFM aligned. This quantity is useful when the condensate dynamics are nonstationary, and time-averaging over a long time window T is more meaningful.

The results in Fig. S10 show two distinct regions of interest. At low powers a single fixed point solution is dominant corresponding to dipole condensates arranged 120° with respect to each other (see insets a-i and a-ii of example solution projected into the spatial domain for clarity), reminiscent of an XY ground state. In this regime there is no vorticity and therefore $\langle M \rangle = 0$. At higher powers we pass the vorticity threshold and vorticity starts growing monotonically. Here, a new family of attractors in which (on-average) two parallel vortices and one antiparallel appear (see insets a-iii and a-iv for example solution). For the given parameters $|J_a| < |J_b|$ the vorticity threshold is associated with clear AFM order as can be seen in Fig. S10B. These results are similar to the observations on coupled nanodisk lasers (see Fig. 2 in Ref. [40]) Notice also that this solution lacks discrete rotational- or mirror-symmetry just like we observe in experiment (see e.g. Fig. 3D in main text).

B. 22-cell system: AFM order in a triangular lattice

We next investigate for AFM order in the finite 22-cell triangular lattice like in Fig. 5 in the main manuscript by solving Eq. (S1). We use the same parameters as in previous section but now fix $\tilde{p} = 1$ which is well above threshold and the vortex bifuraction point in the lattice. The reason why $\tilde{p} = 1$ is sufficient is because of the increased connectivity in the lattice compared to the single triangle (i.e., 6 neighbours in the bulk instead of just 2) which lowers the bifurcation point.

We then repeat the same analysis as we did in the last part of the main manuscript where we look for correlations between the obtained vortex patterns and the configurations of the Ising Hamiltonian [Eq. (1) in the main manuscript]. To do this, we numerically integrated Eq. (S1) in time (over a long enough time interval to capture long-time behaviour) and extracted the average S_z parameter from each cell.

$$\langle S_z^n \rangle = \frac{1}{T} \int_0^T S_z^n \, dt, \tag{S5}$$

We then assigned a binary variable to each cell through the following projection,

$$\sigma_n = \text{sign}\langle S_z^n \rangle. \tag{S6}$$

Repeating this for 1000 random different initial conditions we performed the same analysis of finding the average number of AFM, FM and FREE "spins" and added the result to Fig. 5C in the main text.

SUPPLEMENTARY NOTE 7: INTERFERENCE BETWEEN VORTICES AND OPTIMAL PHASE-RELATION

The relative phase between the vortices is an important degree of freedom as we discuss around the overlap integral, Eq.(11), in the main manuscript. Here, we numerically calculate this integral between two displaced vortices,

$$I_{a,b} = \int \xi(r)^* e^{\mp i\theta} \xi(r') e^{i\theta'} \, d\mathbf{r}. \tag{S7}$$

Here, (r, θ) are the radial and angular coordinate in the cavity plane and $\xi(r)$ are the profiles of each vortex separated by a distance $\mathbf{r}' - \mathbf{r} = d\hat{\mathbf{x}}$. The coordinates of the two pumps are related through $r' = |\mathbf{r}'| = \sqrt{r^2 + d^2 - 2rd\cos(\theta)}$ and $\sin(\theta') = r\sin(\theta)/r'$. Example profiles for counter-rotating vortices are shown in the top panels in Fig. S11. Clearly, the in-phase vortices destructively interfere around the mirror symmetry axis where their overlap over the pumped

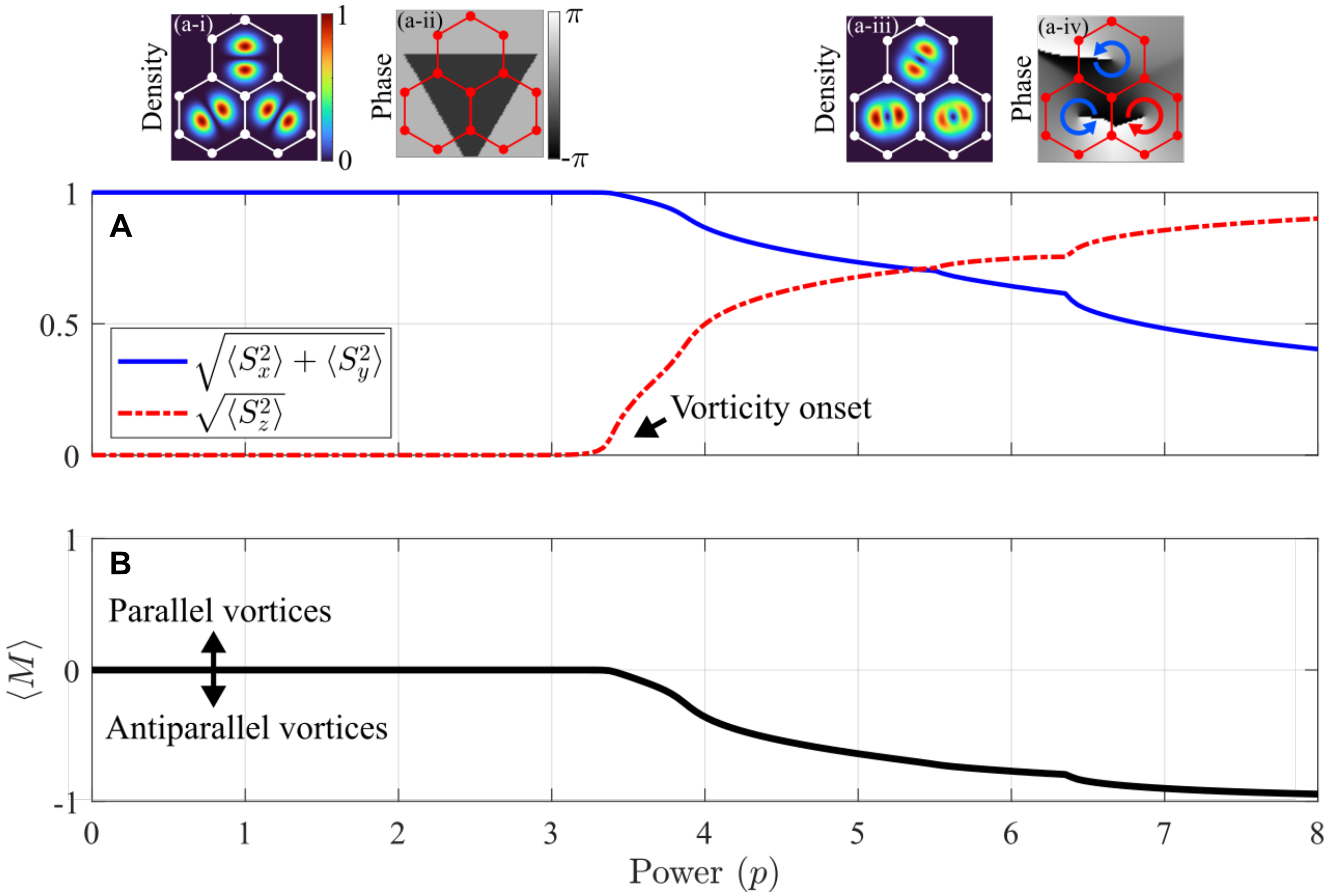

Figure S10. **Comparison of the average condensate population in dipole states ($S_{x,y}$) and vortex states (S_z).** We numerically solve Eq. (S1) over 1000 random initial conditions for different values of the effective power parameter $\tilde{p}$. **a** Shows the time-average and cell-average magnitude of projection onto the $S_{x,y}$ and S_z Bloch vector components. Left and right insets above (**A**) show examples states from simulation for low and high power respectively. (**B**) Corresponding average vortex "magnetism" order parameter. Positive values indicate vortex "ferromagnetism" and negative values vortex "antiferromagnetism".

region is most important. In contrast, the anti-phase vortices constructively interfere which optimizes their mutual gain. This can also be seen from calculating their overlap integral as a function of separation distance (lowest panel) where the larger negative value of the counter-rotating vortices (red curve) implies preference towards anti-phase locking in order to optimize the system gain. This explains why the latter configuration has lowest condensation threshold according to analysis of the eigenmodes of 2 cells and why it is observed in the majority of realizations of such pump landscape in the experiment.

SUPPLEMENTARY NOTE 8: GAIN OPTIMIZATION OF THE VORTEX LATTICE AND ISING ORDER

In what follows we provide an argument for the Ising analysis in the main manuscript from a theoretical point of view. In order to do this we must construct a cost function for the gain between coupled condensates. We will do this based on Eq. (S1) which describes each vortex as a two-component phase-amplitude oscillator in a discrete Gross-Pitaevskii model. The complex-number order parameter for the nth condensate is written $\boldsymbol{\psi}_n \equiv (\psi_{n,+}, \psi_{n,-})^T$, where $\pm$ denote the right-hand and left-hand rotating vortices. In the work (*37*) the phases θ_n of coupled scalar condensates are regarded as a 2D classical XY "spins" $\mathbf{s}_n = (\cos(\theta_n), \sin(\theta_n))$ in the complex plane. Since, in our work, we have a two-component complex state vector $\boldsymbol{\psi}_n \in \mathbb{C}^2$ we need to keep track of both intra- and inter-phases using instead a 4D classical "spin", $\mathbf{s}_n = (a_{n,+}, b_{n,+}, a_{n,-}, b_{n,-})^T$ where $a_{n,\pm}$ and $b_{n,\pm}$ are the real and imaginary parts of $\psi_{n,\pm}$. A straightforward calculation gives us the following energy functional corresponding to the coupling

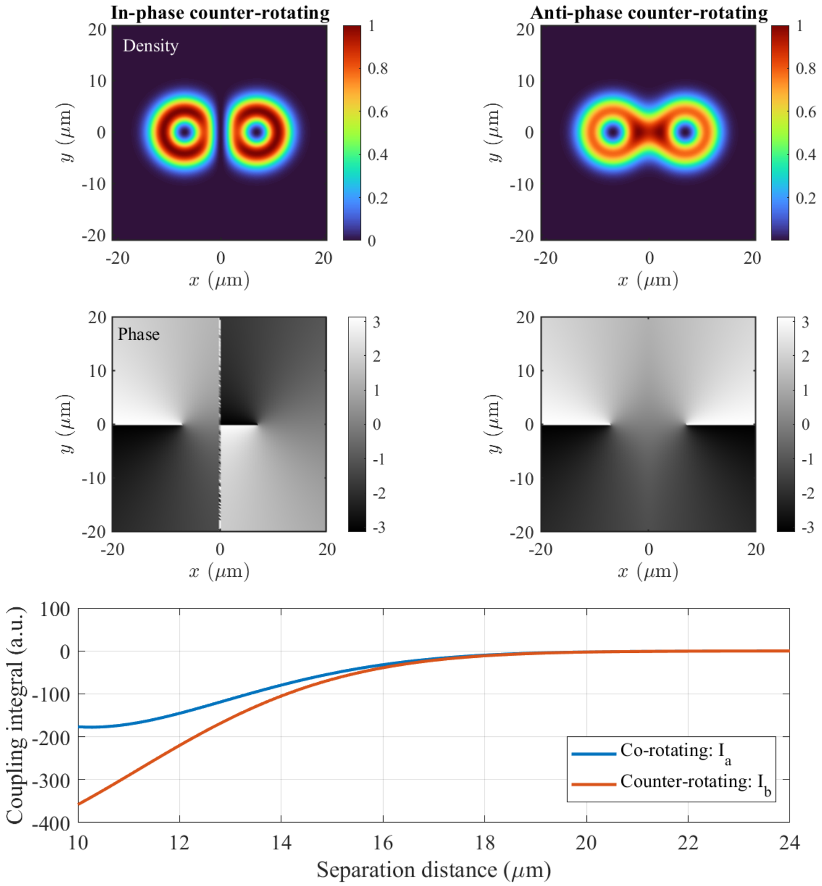

Figure S11. Example superposition of counter-rotating spatially displaced vortex wavefunctions with in-phase (left upper panels) and anti-phase (right upper panels) configuration. Bottom panel shows the coupling integral corresponding to Eq. (11) in the main manuscript.

energy between condensates [last term in Eq. (S1)],

$$\mathcal{J} = \sum_{n,m} J_{n,m}^{\alpha,\beta} \left[(\mathbf{s}_n \cdot \mathbf{s}_m)\delta_{\alpha,\beta} + (\mathbf{s}_n \cdot [(\hat{\sigma}_1 \otimes \hat{\sigma}_0)\cos(2\Theta_{n,m}) - (\hat{\sigma}_2 \otimes \hat{\sigma}_2)\sin(2\Theta_{n,m})]\,\mathbf{s}_m)\delta_{\alpha,-\beta}\right] \tag{S8}$$

Here, $\alpha, \beta \in \{\pm\}$ are the vortex sign indices, the $\delta_{\alpha,\beta}$ is the Kronecker delta function, $\hat{\sigma}_{1,2}$ are the Pauli matrices and $\hat{\sigma}_0$ is the 2×2 identity matrix. The coupling coefficient $J_{n,m}^{\alpha,\beta} \in \mathbb{C}$ is determined by the integrals in Eq. (11) in the main manuscript, and $\Theta_{n,m}$ is the angle of the link between two condensates in the plane. Notice that in the case of a triangular lattice the angles belong the set $\Theta_{n,m} \in \{0, 2\pi/3, 4\pi/3\}$ and are responsible for the geometric frustration.

As we have discussed in the Materials and Methods section of the main manuscript around Eq. (11), the coupling rate between condensates $J_{n,m}^{\alpha,\beta}$ is complex which means that $\mathcal{J}$ is complex. Since the system selects an OAM configuration to optimize the gain then we are interested in maximizing $\text{Im}(\mathcal{J})$. From here on we are only concerned with the complex part of $\mathcal{J}$ and will drop the "Im(.)" notation.

The first inner product term in Eq. (S8) favours co-rotating vortices (FM arrangement). However, this term turns out to be weaker than the second term (see Fig. S11) which instead favours AFM arrangement. For simplicity, we will investigate the case of $\Theta_{n,m} = 0$ corresponding to a non-frustrated linear chain of condensates with uniform coupling strengths $J_{n,m}^{+,-} = J_b$ and $J_{n,m}^{+,+} = J_a = 0$. We then have,

$$\mathcal{J} = J_b \sum_{n,m} \mathbf{s}_n \cdot (\hat{\sigma}_1 \otimes \hat{\sigma}_0)\mathbf{s}_m. \tag{S9}$$

When pumped strongly enough, the presence of dipole states diminishes (i.e., above blue stars in Fig. 4 in the main manuscript) and we can parametrize the state vector as follows,

$$\mathbf{s}_n = (\cos(\theta_n)\cos(\phi_n), \cos(\theta_n)\sin(\phi_n), \sin(\theta_n)\cos(\phi_n), \sin(\theta_n)\sin(\phi_n))^{\mathrm{T}} \tag{S10}$$

Here, $\theta_n \in [0, \pi/2]$ determines the direction and amount of vorticity and $\phi_n \in [0, 2\pi)$ the overall phase at site n. That is, $\theta_n = 0$ corresponds to OAM$= +1$ and $\theta_n = \pi/2$ to OAM$= -1$. Our gain functional then becomes,

$$\mathcal{J} = J_b \sum_{n,m} \sin(\theta_m + \theta_n) \cos(\phi_m - \phi_n). \tag{S11}$$

The cosine term is the XY-Hamiltonian previously pointed out in the work (*37*). The sine term is novel and importantly only depends on the angle θ_n which determines the vorticity in each component. If we project the vortex angles onto their nearest extremes $\theta_n \to \theta_n \in \{0, \pi/2\}$ we can rewrite our gain functional using a binary variable $\sigma_n \in \{\pm\}$ denoting the projected normalized OAM at each site,

$$\mathcal{J} = J_b \sum_{n,m} \frac{1 - \sigma_n \sigma_m}{2} \cos(\phi_m - \phi_n). \tag{S12}$$

We remind that $J_b < 0$ and optimizing (S12), i.e. optimizing the system gain, is the same as minimizing Eq. (1) in the main manuscript because they differ by a sign factor. Notice that the binarized term $1 - \sigma_n \sigma_m > 0$ in the sum is always positive. Therefore, the optimal value is obtained when the "Ising" term is as positive as possible and the cosine term is as negative as possible. This happens for anti-phase $\theta_n - \theta_m = \pi$ and AFM ordered vortices $\sigma_n \sigma_m = -1$, in agreement with our experimental and numerical results.
